# Patients' views and experiences of technology based self-management tools for the treatment of hypertension in the community: A qualitative study

**DOI:** 10.1186/s12875-015-0333-7

**Published:** 2015-09-09

**Authors:** Liam Glynn, Monica Casey, Jane Walsh, Patrick S. Hayes, Richard P. Harte, David Heaney

**Affiliations:** Discipline of General Practice, School of Medicine, National University of Ireland, Galway, Ireland; Discipline of Psychology, National University of Ireland, Galway, Ireland; Clinical Research Facility, National University of Ireland, Galway, Ireland; College of Engineering & Informatics, National University of Ireland, Galway, Ireland; Centre for Rural Health, University of Aberdeen, Inverness, Scotland

## Abstract

**Background:**

Patients with hypertension in the community frequently fail to meet treatment goals. The optimal way to organize and deliver care to hypertensive patients has not been clearly identified. The powerful on-board computing capacity of mobile devices, along with the unique relationship individuals have with newer technologies, suggests that they have the potential to influence behaviour. However, little is known regarding the views and experiences of patients using such technology to self-manage their hypertension and associated lifestyle behaviours. The aim of this study was to explore patients’ views and experiences of using technology based self-management tools for the treatment of hypertension in the community.

**Methods:**

This focus group study was conducted with known hypertensive patients over 45 years of age who were recruited in a community setting in Ireland. Taped and transcribed semi-structured interviews with a purposeful sample involving 50 participants in six focus groups were used. Framework analysis was utilized to analyse the data.

**Results:**

Four key inter-related themes emerged from the analysis: individualisation; trust; motivation; and communication. The globalisation of newer technologies has triggered many substantial and widespread behaviour changes within society, yet users are unique in their use and interactions with such technologies. Trust is an ever present issue in terms of its potential impact on engagement with healthcare providers and motivation around self-management. The potential ability of technology to influence motivation through carefully selected and tailored messaging and to facilitate a personalised flow of communication between patient and healthcare provider was highlighted.

**Conclusions:**

Newer technologies such as mobile devices and the internet have been embraced across the globe despite technological challenges and concerns regarding privacy and security. In the design and development of technology based self-management tools for the treatment of hypertension, flexibility and security are vital to allow and encourage patients to customise, personalise and engage with their devices.

## Background

Newer technologies such as mobile devices and the internet are ubiquitous in modern society. They have led to many examples of mass behaviour change in relation to everyday tasks such as banking, shopping, communication and information gathering. Health related behaviour change driven by such technologies is a more recent phenomenon yet has grown exponentially in recent years with downloads for health and lifestyle related Mobile Applications or “Apps” expected to exceed 25 billion in 2015 and 50 billion in 2017 [1]. Hypertension is an important public health problem in terms of associated stroke and cardiovascular events. However, blood pressure goals are achieved in only 25–40 % of the patients who take antihypertensive drug treatment [2, 3], which is something that has remained relatively unchanged for the last 40 years [4]. The most recent Cochrane review of non-pharmacological interventions to control blood pressure confirms the benefit of self-monitoring, organisation interventions and appointment reminder systems [5]. Indeed, use of self-monitoring of blood pressure by patients and professionals has gained popularity and is now recommended in certain patients in national and international guidelines [6] while meta-analyses of randomised trials on the subject suggest a benefit in terms of mean blood pressure and blood pressure control [7, 8]. It is evident that such non-pharmacological interventions to improve control of blood pressure can be organised and facilitated by newer technologies, particularly mobile devices such as smartphones, 1.25 billion of which were sold to end users in 2014 [9]. In addition, the powerful on-board computing capacity of mobile devices, along with the unique relationship individuals have with newer technologies, suggests that they have the potential to influence behaviour. The potential of technology generally, and mobile devices in particular, to influence human behaviour is due to the strong attachment people have to their mobile devices, the multi-use capability of such devices and the fact that they are carried wherever they go [10]. People tend to interact with, or check, their mobile devices regularly and this repeated reviewing or ‘checking habit’ is reinforced by immediate visible information, rewards and in some cases entertainment in a “gaming” environment [11]. This has the potential to encourage and facilitate the individual patient to use technology to improve and manage their health on an ongoing basis. In relation to self-management, there is some emerging evidence that mobile devices are effective in promoting physical activity [12, 13] and some evidence about patient perspectives and the mechanisms by which these applications might promote behaviour change has also emerged [14]. However, little is known regarding the views and experiences of patients using such technology to self-manage their own hypertension and associated lifestyle behaviours. The aim of this qualitative study was to explore patients’ views and experiences of using technology based self-management tools for the treatment of hypertension in the community.

## Method

### Recruitment of interview participants

This qualitative study was conducted with known hypertensive patients over 45 years of age who were recruited in a community setting in Ireland. Patients were recruited through a snowballing technique [15] which sought to identify a purposeful sample of patients with hypertension of varying ages; gender; socioeconomic status; geographic location (rural and urban); employment (working and not working or retired); time of diagnosis (within past 12 months and longer); on and not on medication; with and without multimorbidity; and those of high, medium and low technology literacy (Table 1). Even though hypertension has not been shown to be a gender sensitive issue, there is evidence that the use of technology can be quite gender specific [16]. Therefore groups were segmented into single gender to facilitate a free-flowing discussion particularly in relation to use of technology [17]. As is commonplace in qualitative research, an iterative approach was taken in order to be responsive to, and incorporate, findings from the data as they emerged [18]. Recruitment continued until data saturation was reached, at the point where new data collection did not shed any further light in this issue investigation [19] and no new themes emerged. Ethical approval was granted by the Clinical Research Ethics Committee, Galway University Hospitals (reference number CA 1220; 26th February, 2015) and all participants provided written informed consent.

**Table 1:** Focus group sampling characteristics

Group 1	Males, 45–60 years; urban; mix of working and not working or retired; range of when diagnosed with hypertension (< and >12 months); mix of those who take medication and those who don’t; some with other medical conditions, some without; mix of those with medical card and those without; mix of participants of high, medium and low technology literacy.
Group 2	Females, 60+ years; urban; mix of working and not working or retired; range of when diagnosed with hypertension (< and >12 months); mix of those who take medication and those who don’t; some with other medical conditions, some without; mix of those with medical card and those without; mix of participants of high, medium and low technology literacy.
Group 3	Females, 45–60 years; rural; mix of working and not working or retired; range of when diagnosed with hypertension (< and >12 months); mix of those who take medication and those who don’t; some with other medical conditions, some without; mix of those with medical card and those without; mix of participants of high, medium and low technology literacy.
Group 4	Males, 60+ years; rural; mix of working and not working or retired; range of when diagnosed with hypertension (< and >12 months); mix of those who take medication and those who don’t; some with other medical conditions, some without; mix of those with medical card and those without; mix of participants of high, medium and low technology literacy.
Group 5	Males, 60+ years; urban; mix of working and not working or retired; range of when diagnosed with hypertension (< and >12 months); mix of those who take medication and those who don’t; some with other medical conditions, some without; mix of those with medical card and those without; mix of participants of high, medium and low technology literacy.
Group 6	Females, 45–60 years, rural mix of working and not working or retired; range of when diagnosed with hypertension (< and >12 months); mix of those who take medication and those who don’t; some with other medical conditions, some without; mix of those with medical card and those without; mix of participants of high, medium and low technology literacy.

### Focus groups

**Fig. 1:**
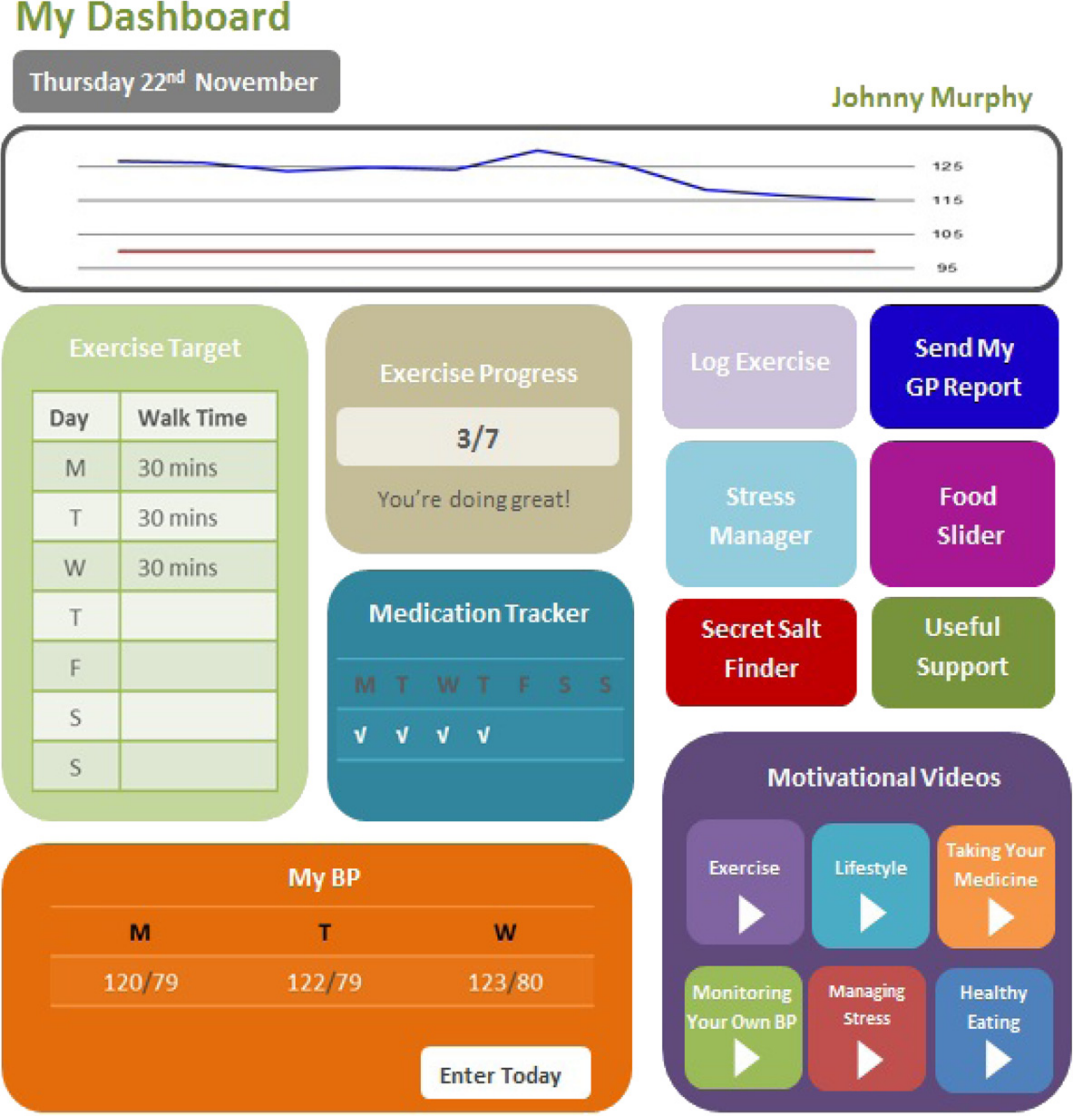
Web-based “Dashboard” concept used in focus group discussion

The topic guide focus group questions were developed by reviewing other qualitative research exploring use of technology in the management of hypertension and behaviour change to develop a sense of what questions might elicit the most informative answers. These were then discussed with the research team and lay technology users to decide what questions would most thoroughly explore the participants’ experiences. The participants were consented for interview, audio recording and use of anonymous quotations. The focus groups were held in a neutral venue and were conducted by qualitative researchers who were independent of the study research team. The final topic guide is described in Table 2 and included exposure to a web-based concept for self-management of blood pressure (Fig. 1). To enhance reliability, all interviews were recorded and transcribed verbatim by an independent professional transcriber and the audio files and transcriptions were compared [20].

**Table 2:** Interview topic guide

The focus group began with an introduction on the study with some background information provided about high blood pressure (with the term hypertension being introduced and defined) and self-management
1. Where did you hear about the study—did you volunteer or were you recruited?
2. What was your initial reaction when you were asked to join?
3. Were you looking forward to it and why? (If not, why not?)
4. When was your hypertension diagnosed and how is it managed?
5. Do you know a lot about hypertension and if so, where have you found out this information?
6. What do you do yourself to manage your hypertension?
7. Do you use technology such as mobile phones, the internet, Apps in your everyday life?
8. How do you find working with such technology?
9. Do you find such technologies useful and if so for what?
10. Do you have concerns or difficulties in your use of technology in your everyday life?
11. Do you use such technologies in your everyday life for managing your health? (examples: physical activity tracking, medication reminder, diet tracking and advice?)
12. Do you use such technologies in your everyday life specifically for managing your hypertension?
13. Did you have any other strategies besides technology for managing your hypertension?
14. How did you feel about letting people around you know that you were trying to manage your hypertension?
15. Would you be interested in being part of an online or face to face forum with other people with hypertension to share tips, and motivate and help each other?
16. Is there anything else that you think would be helpful or motivating to you in managing your hypertension?
17. You are now going to be shown an example of a web-based tool (Fig. 1) which would be available on your mobile device or computer which is being designed to help people manage their own hypertension. What is your initial reaction to such a tool?
18. Are there aspects of the tool that you would find useful and you might use?
19. Are there aspects of the tool that you would definitely not be interested in?
20. Why do you think such a tool might be useful to you?
21. Would you have any concerns about using such a tool?
22. What is the best thing about this tool?
23. What improvements would you like to see to the tool to improve its benefits?
24. Would you consider using social media i.e. Facebook or Twitter etc. to discuss your hypertension?
25. What role will managing your hypertension play for you in that future?
26. Are there any further issues you would like to discuss?

### Analysis

The five stages of the Framework Process were followed in the examination of the qualitative data which included familiarization, thematic framework identification, indexing, charting, mapping and interpretation [21]. Coding was conducted with another researcher from a different professional background for inter-coder reliability [22]. To heighten reflexivity, four members of the research team, (a nurse, a general practitioner, a clinical engineer and a psychologist) reviewed all the data and contributed to the thematic analysis [23]. NVivo 10 software [24] was used to organize and code the transcripts to facilitate the analysis and comparison of relationships between the coded ideas [25].

## Results

### Participants

In total, 237 participants were screened and 57 were asked to take part in the focus group interviews, of which 50 (88 %) agreed, consented and participated. The focus group sampling characteristics are described in Table 1. Interviews were semi-structured and were conducted by one researcher with the addition of a second researcher for quality control purposes. Participant characteristics are shown in Table 3.

**Table 3:** Characteristics of focus group participants *n* = 50

Characteristic	
Mean age, years (range)	59 (46–73)
Female, *n* (%)	25 (50)
Medical Card, *n* (%)	26 (52)
Urban, *n* (%)	24 (48)
Technology literacy	
Internet at home, *n* (%)	49 (98)
Smartphone, *n* (%)	47 (94)
Email on phone, *n* (%)	43 (86)
Downloaded apps previously, *n* (%)	44 (88)
Clinical characteristics	
Taking medication	29 (58)
Multimorbidity	25 (50)

### Key themes

The themes that emerged from the data were classified into four major interrelated themes:

- Individualisation
- Trust
- Motivation
- Communication

### Individualisation

The first major theme that emerged from the qualitative focus group data was the theme of “individualisation”. This refers to many different aspects of the patient’s approach to the management of their hypertension specifically and their health generally. Firstly, patients had developed distinct approaches to knowledge gathering and knowledge generation in relation to their hypertension specifically and their health generally.

*“So you have to make your way through seeing what is right and what is wrong and I would be of the impression that if something is said maybe 3 or 4 times on different websites then there is a certain logic that might be correct you know. So I will follow down that line in my line of thinking until something else proves what I am seeing there.”*

*“It is an app….it just comes up… it is actually on my e-mails …….but it is amazing what you get from it….so many different things that you could take from that you know ….. I gave up cigarettes and it gives you all different things.”*

Secondly, they demonstrated very different approaches to health-seeking behaviour.

*“I see the guy every 6 months and I don’t worry about it until then right….”*

*“About 3 or 4 years ago there I went to the doctor just as part of a general check-up and he said your blood pressure is border line. So at that stage I kind of decided o.k. there is history of diabetes in the family as well so I was kind of conscious of that as well. So in that respect then I started doing more exercise, dropped a bit of weight and I more or less cut out the alcohol as well, changed the food I was eating.”*

Thirdly, they described often quite singular approaches to how they managed their chronic disease or diseases as well as general measures in lifestyle and risk factor reduction, which did involve at times rejecting or not complying with medical advice.

*“And then they were talking about walking helping the blood pressure….I’d only kind of have been diagnosed with it…I wasn’t that long on the medication …… so I came off it and went walking….and I’ve haven’t told the doctor yet …”*

*“It is difficult because it is not something I am particularly conscious about. Because there is little or no symptoms it is not something that you would be thinking about so the fact that you are on pills for it even puts you in a position where you kind of forget about it totally because you assume that the pill is keeping it under control. So I would hate for instance to know what my blood pressure was at any given moment sort of thing you know. I would be going oh my God my blood pressure is up…..”*

### Trust

The second major and interrelated theme was the issue of “trust”, as this underpinned many of the interactions that individual patients had with their healthcare provider, their healthcare tools including medication and their healthcare information. The traditional paternalistic model and doctor-centred approach to the interaction with the healthcare professional appeared to be very much based around the principle of trust.

*“It is almost like you want to forget about it and just get on with your life and trust that the pills that you are taking are controlling it. So going to the doctor every so often and him taking my blood pressure and it being o.k. that’s fine by me I don’t really want to know any more about it.”*

*“…there is something psychological about going to the doctor….somebody telling you… I wouldn’t trust (my blood pressure) for 6 months without going to the doctor.”*

This approach suited many patients, while others described examples of this trust breaking down or feelings of vulnerability in relation to the conventional tools of the medical approach such as medication itself or the patient-healthcare provider consultation.

*“My doctor recommended that I get one (a blood pressure monitor) and the week after I was going to the consultant and I said what do you think of this and he said you would be stressed doing it.”*

*“When I was taking the blood pressure on my own monitor at home…. I would bring them into to (my doctor) to give him an idea…….. I kind of got the impression that it was kind of, well I’m taking it now here and this is the right one, you know.”*

Many examples of alternative strategies used to cope or to replace the conventional tools of the medical approach were also described such as home monitoring.

*“I don’t know if my blood pressure is high or low I have no idea so all I was trying to do was understand because I knew every time I went to the doctor it was up because as soon as you see the doctor you go….he’s taking out the blood pressure thing he is going to say it is up…. so by doing it in a more relaxed setting at home I just wanted to see would it be different and it was. It was substantially different and it appeared to be much lower than every time I went to the doctor.”*

These strategies often involved other sources of information and advice such as other perceived “experts” or expert patients; tools for self-management such as home blood pressure monitoring and web-based information sources However, the most common concern described in relation to these alternative strategies was the issue of trust. This centred on concerns about accuracy of information on the internet, security of data and data transfer and accuracy of “off the shelf” technology such as blood pressure monitoring machines or smartphone applications.

*“We all have doubts about the quality of the information we are getting so we are looking for something with real good quality verifiable information that is easy to gather but it is actually exact.”*

*“And you have the mobile testing clinics and they have got a result in there and then I have gone to the doctor and the results are relatively different so you often wonder.”*

One of the most surprising and striking trust issues came in relation to the patient’s own beliefs around engagement and effectiveness. Many patients openly doubted their own ability to engage in behaviour change but this became understandable in light of the lack of belief that they placed in the effectiveness of lifestyle change to improve their chronic disease status or their health and well-being.

*“…no matter how much walking and exercising you do and low salt food you eat your going to end up still having high blood pressure so you have to have medication for the rest of your life and that’s it.”*

### Motivation

The third major theme described the issue of “motivation”, particularly in relation to patient self-management. It became obvious that motivation was multi-factorial and was made up of separate building blocks which were inter-dependent and sometimes sequential in their relationship to one another. Patient’s level of knowledge and ability to generate knowledge for themselves affected their attitude to their condition.

*“My mother died of a stroke in her 70’s, so it was in the family, so actually what I did was I saw a blood pressure monitor and I bought it, and I discovered (my high blood pressure) myself, and I went to my GP and he told me that it was only, not to worry about it but it started going up…I was exercising quite a bit and I wasn’t over weight really, at the time, so he put me on medication then, because of the history…the medication did the trick really. But I take my own blood pressure all the time you know….I would I take it (my blood pressure) once a week at least….I could take mine at home now…and you go up to (my doctor) and it’d be higher…… so, it’s difficult like, you know, you’d have to get an average over the day, you know.”*

This attitude was often based on a perception of risk which appeared to influence the timing and frequency of their health seeking behaviour and their active engagement with self-management.

*“So it is just a change of lifestyle I don’t always stick to it but … my Dad had stroke in his early 60s so things like that you eventually start thinking about it well it’s not too far away do you know what I mean.”*

It is possible to distil this concept into the following statement: knowledge and attitudes predicted motivation, motivation predicted intention and intention then predicted behaviour.

*“Well it’s once a week now I (measure my blood pressure), I was doing it I had actually a chart because I done it for the 3 weeks, and I could see it coming right down, and down, and down, and feel myself getting better, so this is why I’m really chuffed now, because for years I haven’t felt really good and now I feel great.”*

This could be described as a process of “action-planning”, where the potential advantages of newer technologies were evident in their ability to create the building blocks described above.

*“You can look back at it and it gives you motivation it can help. There are mornings you will wake up in the morning and you will look at your tablet and you don’t even want to take them. You just look at them and you go I am sick of this. But you do and then I suppose if you do yourself a small plan you are achieving it and then that gives you motivation and stuff.”*

Patients described a continuous journey of success and failure where helpful aspects of technology such as information, feedback, reward systems and automaticity were attempting to embed new habits in relation to self-management.

*“My GP then sent me to a dietician that time, that was helpful as well you know, kept to that for about a year or two or three and I wandered away from it again….the information on high blood pressure, again I would go to the internet to remind myself about it, you know, the only thing I’ve kept good at is the low intake of salt you know.”*

*“Because of, my good lifestyle has gone fallen by the wayside..… and you know I’m travelling a lot and …. I know its am, so you know yourself, it’s getting yourself back in to it something, I bought a bicycle I haven’t collected it yet.”*

The concept of “two steps forward and one step back” was also described frequently which recognised the fact that there were many factors that could interrupt or disturb an action plan and thus “coping” or “contingency planning” was an important ability in self-management.

*“I suppose things like holidays and Christmas and that. They are the times that people wouldn’t be watching what they eat or drink and things like that. You would be less conscious of it and you would tend to take your foot off the pedal a bit you know. But then you try and make up for it. As I said when everybody else is over indulging its hard sometimes to stick to that regime you know.”*

### Communication

The importance of communication as the basis of the relationship between the healthcare provider and patient was widely recognised.

*“I get my GP to check it, he checks it every 6 months for me. But I also have a first aid doctor at work as well he is very good and he will do it voluntarily. Every month or so you know.”*

*“Another source for me would be pharmacists. He encouraged me to borrow his for a week and chart it and bring it back to him and he looked at it and he said that is high that is low or that’s ok there is your pattern keep taking the tablets. So I did but more so than just relying on the doctor. You see the pharmacist more often then you see the doctor.”*

Many other sources of communication were also described as influencing the patients approach to their hypertension specifically and their health generally. These included information from the internet, medical devices and advice and feedback from peer groups and family members.

*“I have the Web MD. It just sends you an update every day, it is like a front page of their site would come up every day….there is a search site on the site and you can just tap in if you were looking for information anything from as I said change of life or giving up cigarettes or high blood pressure or anything at all.”*

However, a need for personalised communication from a reliable source be it doctor, nurse, pharmacist or peer was also described:

*“And make it relative because when you come away from the doctor the first time you know your (blood pressure) figure. I know over time we can lose interest in the figures because we take the tablets and guess what it is not that interesting anymore. But when you go in those first few times we all knew the figure.”*

*“Calibrate it….if you do this you are likely to bring your blood pressure down to that. I am not sure if that is a simple thing that can be done, but some sort of rough guide into what’s what.”*

The potential for technology to remove the need for, replace or facilitate communication between these different sources was also described.

*“The thing is when I got my bloods done last week. I get them done very early in the morning so the nurse said ok we will send them out in the post and I said no send them back by e-mail….”*

The traditional model or one-way flow of information from healthcare provider to patient was challenged with the ability of technology to provide, store and create a two-way flow of communication also suggested.

*“I don’t think the Doctor would be very impressed with me…having all the information kind of like, you know….I wouldn’t see her to be happy to be getting a report sent to her. I just have a feeling that she would feel that she was being undermined, you know, I just, you know I would.”*

The potential of technology to improve the quality and variety of information received was recognised in addition to facilitating the flow of information back to the healthcare provider.

*“So I would like to be able to …screen save it ….scan it…and save it and bring it up and show it to (my doctor)….”*

It was felt that this potential created the possibility of fundamentally changing health seeking behaviour if carefully selected and tailored messaging was used:

*“If you got a text on a Monday, Wednesday and a Friday, did you take your “A..B..C” today or whatever you know, that’s it, like simple, did you exercise today question mark and you could put in “yes” …it has to be individually tailored.”*

## Discussion

### Summary of main findings

Health related behaviour change driven by technology is a relatively recent phenomenon, but appears to have the potential to positively transform, in a unique way, user’s self-management of hypertension and other lifestyle behaviours. The data from this study suggests that in design and development of interventions to enhance self-management of hypertension and lifestyle behaviour, it is vital to not take a “one size fits all” approach. Rather, it is important to build in enough flexibility in the system or intervention to facilitate and indeed encourage individual patients to tailor, personalize and prioritise their approaches to self-management. In addition, the issue of “trust” was striking in terms of how it underpinned many of the interactions that individual patients had with their healthcare provider, their healthcare tools including medication and their healthcare information. Fundamentally, this absence or lack of trust seems to be an ever present issue in terms of its potential effect on engagement with healthcare providers and motivation around self-management. Therefore, in relation to the introduction of a new technology or platform for engagement, it is crucial that every effort is made to alleviate patient concerns in this area and create confidence quickly in terms of quality and security. Meanwhile, the multi-factorial and complex nature of motivation was elucidated as was the potential ability of technology to provide information, feedback, reward systems and automaticity which could embed new habits in relation to self-management. The potential for technology to facilitate a personalised flow of communication between patient and healthcare provider was recognised as was the ability of technology to contribute to a hierarchy of motivation through carefully selected and tailored messaging.

### Comparison with existing literature

**Fig. 2:**
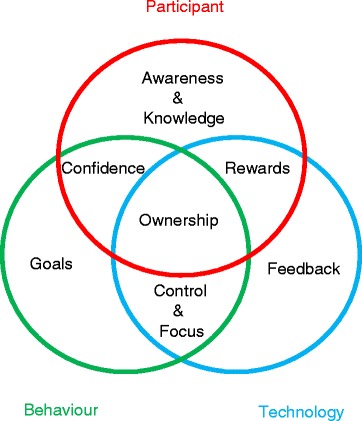
The “Know-Check-Move” effect

Employing theories applicable to health behaviour change can support efforts to harness technologies to promote lifestyle change [26]. However, investigation of the interactions between users and such persuasive technologies requires much greater elucidation. The identification of the person’s needs and the integration of their personal goals have been shown to be important for initiating and executing the change process. This individualisation is also important due to differences in health seeking behaviours that are well evidenced in the research literature. Miller characterises people as either “monitors” or “blunters” in the face of perceived threats to their health [27]. Monitors are highly attentive and sensitized, and tend to amplify threats, whereas blunters avoid and minimize the same threats. Understanding the effects of individual coping styles on patient adaptation can help physicians increase compliance. Health behaviour change interventions are more effective when they are grounded in psychological theory and draw upon behaviour change techniques. Health behaviour and self-care requires active decision making and self-regulation. It has been recommended that interventions should include behaviour change techniques, and also clearly define these techniques. For example, the COM-B model of behaviour change includes the necessity of three components in order for behaviour change to occur, namely: opportunity, capability, and motivation [28]. In this system, capability can influence motivation and behaviour, and behaviour can also influence both of these factors which was also illustrated in the data from this study. Our study has provided some data around the key issues for patients in relation to the use of such innovations for chronic disease management and lifestyle change. The issue of trust which our data has highlighted is perhaps one reason for the relative success of nurse and pharmacist interventions in the management of high blood pressure [29] as generally, patients will have been interacting with these individuals over a long periods of time and will have built up high levels of trust with them as a result. This, in turn, is likely to lead to higher levels of engagement with the medical advice provided. The building blocks evident in our data around motivation towards behaviour change contain many of the elements already described in the literature as the “know-check-move” effect (Fig. 2) [14]. This describes how technology can affect lifestyle behaviour change in a transformational way. The adoption of new technology is also not just dependent on motivation to change a lifestyle or behaviour. In traditional technology acceptance models, factors such as previous technology experience, social support and perceived usefulness significantly influence whether a potential user adopts a technology. Telling somebody that a technology will benefit them does not necessarily mean they will perceive it that way. This is particularly true if they have limited (or negative) previous experience with similar technology or do not receive continued support from social peers when using and learning the new technology [30]. As changes in technology become more fluid and competition in the App market increases, factors such as perceived ‘ease of use’ and perceived complexity are having a greater influence on technology adoption [31]. This factor becomes particularly important when dealing with older adults, a population group who lag behind the general population in terms of technology adoption. Greater demands on sensory, cognitive and psychomotor capabilities can lead to immediate rejection of a technology, particularly those with challenging interfaces [32]. This rejection can occur within minutes of using a technology for the first time [33]. Therefore when designing technology which requires high levels of interaction between user and interface, designers must ensure that the demands of the interface do not exceed the capabilities of the user. The most effective way to achieve this is to apply structured human centred design cycles which involve the end user at different stages in the development process, through the use of usability testing techniques such as focus groups, use case analyses, inspections and structured user testing [34]. These techniques can be applied to the development of websites, mobile apps and physical devices for blood pressure management. Helpful aspects of technology such as information, feedback, reward systems and automaticity have the potential to embed new habits in relation to self-management. However, patients describe a continuous journey of success and failure in their relationship with newer technologies. It may be that the timing of such interventions is critical and their effect most significant in certain cohorts of patients such as newly diagnosed patients, or those with poor concordance or sub-optimal control.

### Strengths and limitations

This study provides original data regarding patients’ views and experiences of using innovative technologies for the promotion of behaviour change and self-management around hypertension. Additional strengths of this study included the number of focus groups (*n* = 6), the total number of participants (*n* = 50) and the comprehensive sampling procedure. This was felt to be a pragmatic and ‘real life’ exploration of the use of this technology in a realistic setting which should improve external validity of the study data. Reflexivity was heightened by the multidisciplinary research team reviewing the data, but this may also be seen as a limitation as they may have taken a different emphasis from that of an independent observer. In addition, the sampling frame limited participants to hypertensive patients so data may be different in the context of the challenges of other chronic diseases such as diabetes or cardiovascular disease.

## Conclusions

Newer technologies such as mobile devices and the internet have been embraced across the globe despite technological challenges and concerns regarding privacy and security. This process has triggered many substantial and widespread behaviour changes within society. Despite this users are very individual about their use and interactions with such technologies. This study has demonstrated that technology has the potential to trigger a complex yet engaging behavioural change process for patients for the management of hypertension, hence enabling individuals to take ownership of their own health and healthcare at a time and place of their own choosing. Even in terms of the same chronic disease (hypertension) and similar risk factors (smoking, alcohol, obesity, exercise), patients develop very individual approaches to prioritisation which often depended on personal or contextual factors rather than disease specific factors. These individual differences further serve to highlight the importance of identifying the patient’s profile of barriers to action, and then being able to tailor the intervention approach accordingly. In addition, due to the novel nature of the technology, it can provide a neutral space in which patient and healthcare provider can discuss and negotiate a management plan around often challenging issues such as concordance, sub-optimal control and lifestyle change. The flexibility and inherent motivational ability of newer technologies seems to have the potential to respond and improve the ability of patients to cope with the vagaries of normal life thus leading to an increase likelihood of sustained behaviour change. In relation to episodes of dis-engagement, the presence of such a platform on a mobile device means that the potential for re-engagement is only a “click” or a “swipe” away. This is particular true if patients have quality information at their fingertips which is tailored, personalized and prioritised by the patient themselves.

## Abbreviations

App: Application
NVivo: Qualitative data analysis software
GP: General Practitioner or family doctor
WebMD: Website for medical information and diagnosis
Email: Electronic mail
COM-B: “Capability, Opportunity, Motivation and Behaviour” model of behaviour change
